# Posterior Reversible Encephalopathy Syndrome and Pre-eclampsia/Eclampsia: Anesthetic Implications and Management

**DOI:** 10.7759/cureus.23659

**Published:** 2022-03-30

**Authors:** Andrea R Trent, James W Parry, Jordan E Yokley, Kurt W Grathwohl

**Affiliations:** 1 Anesthesiology, Brooke Army Medical Center, Fort Sam Houston, USA; 2 Medicine, Uniformed Services University of the Health Sciences, Bethesda, USA

**Keywords:** mri brain and spine, anesthesia neurotoxicity, pregnancy-induced hypertension, obstetric anesthesia, preeclampsia-eclampsia, posterior reversible encephalopathy syndrome (pres)

## Abstract

Posterior reversible encephalopathy syndrome (PRES) is a rare neurologic disorder that has recently become more frequently diagnosed. While the exact etiology of PRES remains unclear, multiple diseases are associated with PRES. Moreover, there is increasing recognition of the association of PRES in pre-eclampsia/eclampsia with advancements in imaging techniques and increased awareness of the disorder. While pre-eclampsia/eclampsia alone presents unique perioperative challenges, PRES further complicates anesthetic management. Unfortunately, the anesthetic management for these critically ill and complex patients is not well elucidated and it is unclear whether the anesthetic choice may actually worsen neurologic symptoms. We describe two different presentations of PRES with pre-eclampsia/eclampsia, their anesthetic implications, and management.

## Introduction

This article was previously presented as a meeting abstract at the 2022 SOAP 54th Annual Meeting on May 15, 2022.

Posterior reversible encephalopathy syndrome (PRES) is a rare neurologic disorder that is gaining awareness as imaging modalities improve and recognition increases. It is considered a diagnosis of exclusion that lacks clear diagnostic criteria but is typically characterized by hypertension, acute onset of neurologic symptoms, focal subcortical vasogenic edema on neuroimaging, and reversibility of imaging findings [1]. The incidence in specific susceptible population subsets (pediatric intensive care, end-stage renal disease, systemic lupus erythematosus, and solid organ transplantation) is <1% with the overall incidence difficult to determine and likely under-recognized [2,3]. Conditions associated with PRES include hypertension, sepsis, renal insufficiency, cytotoxic medications and immunosuppressive therapy, and autoimmune disorders [3]. However, there is increasing evidence that pre-eclampsia and eclampsia are associated with the diagnosis of PRES when neurologic symptoms are present [2,4]. The anesthetic management of patients with pre-eclampsia/eclampsia is complicated by multiple physiologic disturbances; however, little is known about anesthetic implications in concomitant PRES. Some case reports implicate the anesthetic technique as the cause of PRES in otherwise healthy surgical patients [5-7]. More information is needed on these overlap syndromes in order to determine optimal anesthetic care for this unique subset of obstetric patients.

## Case presentation

Case 1

**Figure 1:**
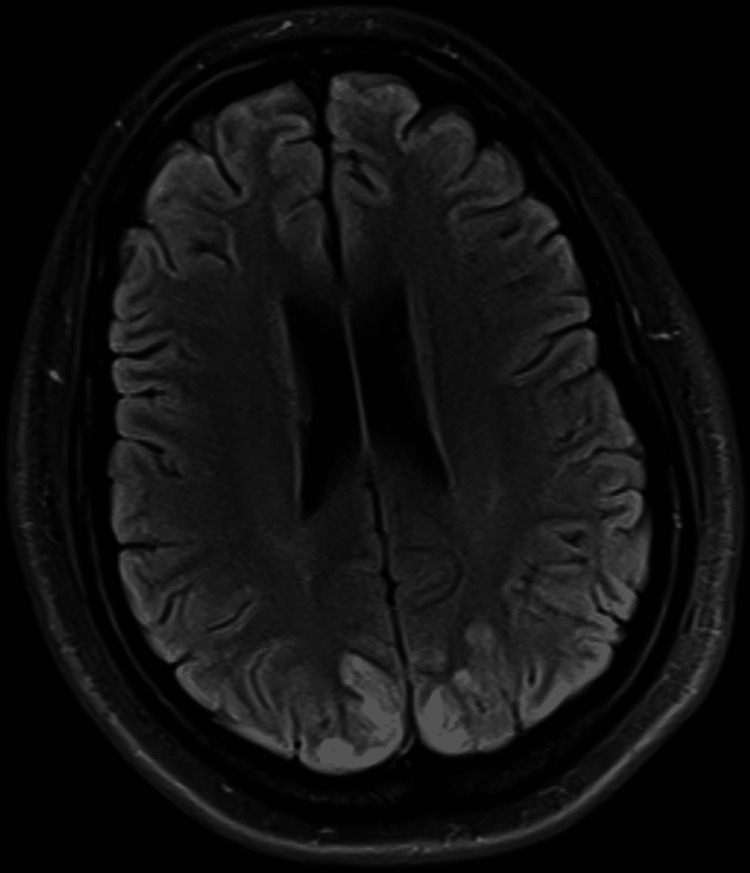
MRI demonstrating increased T2 and FLAIR signal in cortical and subcortical regions of the parietal and occipital lobes.

A 23-year-old female at 24-week-gestation presented to the hospital complaining of bilateral vision loss, headache, nausea, and vomiting. Her initial blood pressure was 210/110mmHg for which she received labetalol, hydralazine, and nifedipine, as well as intravenous magnesium sulfate for seizure prophylaxis. Past medical history included stage-3 chronic kidney disease secondary to focal segmental glomerulosclerosis. The patient was co-managed by her nephrologist and did not require antihypertensive therapy before or during the pregnancy. Physical exam was notable for right homonymous hemianopia with an otherwise unremarkable neurologic exam and mild bilateral pedal edema. Laboratory evaluation demonstrated significant worsening of renal function (baseline creatinine 2.0mg/dL, now at 3.6mg/dL), elevated liver-associated enzymes, uremia, thrombocytopenia, anemia, and proteinuria. Magnetic resonance imaging (MRI) of the brain without contrast revealed symmetric cortical and subcortical signal abnormality of the posterior parietal and occipital lobes (Figure 1). As a result of the clinical presentation and imaging, she was diagnosed with early-onset pre-eclampsia, HELLP (hemolysis, elevated liver enzymes, and low platelets) syndrome, and PRES. After reducing her blood pressure below 140/80mmHg, the patient’s headaches and visual symptoms resolved. Despite resolution of symptoms, laboratory evaluation demonstrated persistent renal injury and HELLP syndrome. Given the severe features of pre-eclampsia and to reduce the risk of maternal and fetal complications, the obstetric team recommended cesarean delivery. Considering the patient’s mild thrombocytopenia (128,000/µL), uremia, and potential for disseminated intravascular coagulation, the team obtained a thromboelastogram (TEG). The TEG subsequently demonstrated normal coagulation dynamics. After discussing the risks and benefits of anesthetic options with the patient, the anesthesia team performed a subarachnoid block using 12mg bupivacaine and 150µg morphine. A premature 24-week infant was delivered without incident and admitted to the neonatal intensive care unit. The patient experienced no adverse anesthetic complications, and her renal and hepatic function returned to baseline over the following week.

Case 2

A healthy 18-year-old female in the third trimester was transported to the emergency department (ED) by ambulance after a witnessed generalized tonic-clonic seizure at home. The patient had no medical problems or complications with pregnancy prior to this presentation. She initially complained of headaches for the preceding few days and then developed “visual changes,” confusion, and difficulty with activities of daily living. Shortly thereafter, the patient developed “shaking” of the upper extremities and loss of consciousness. She had several more generalized tonic-clonic seizures en route to the hospital and at presentation. A rapid evaluation demonstrated regular respiratory rate and oxygen saturation, blood pressure of 160/100mmHg, and a Glasgow coma score of 8 (no eye opening to painful stimulation, moaning, and localizing to pain). A fetal assessment revealed a heart rate of 140-160 beats per minute, cephalic presentation, and size consistent with 37-week-gestation. After establishing intravenous access, the patient received magnesium and several doses of labetalol. Blood pressure improved to 130/80mmHg, and tonic-clonic activity ceased, though the patient remained post-ictal. A head computed tomography (CT) demonstrated no acute intracranial pathology, including masses or bleeding. Laboratory data demonstrated severe lactic acidosis (15.5mmol/L), pH of 6.8, mild elevation of liver-associated enzymes, and significant proteinuria. Complete blood count (CBC) was unremarkable; however, repeat evaluation six hours after admission was notable for thrombocytopenia (46,000/µL). In consultation with neurology, the patient continued magnesium therapy for seizure prophylaxis and received a brain MRI, which demonstrated cortical and subcortical signal abnormality in the bilateral parietooccipital/frontotemporal lobes and a small left parietal hemorrhage. Based on these findings, she was diagnosed with PRES. The obstetric team recommended urgent delivery with preference of cesarean over vaginal delivery due to risk of exacerbating intracranial hemorrhage with vaginal delivery. Due to the severe metabolic abnormalities, hemodynamic issues, mental status changes, significant thrombocytopenia, HELLP syndrome, and the potential for severe coagulopathy, the anesthesia team performed the delivery under general anesthesia with induction using standard doses of propofol and succinylcholine and maintenance with 2.0% sevoflurane. The anesthetic was uncomplicated, and the infant was delivered with APGAR scores of 8 and 9 at one and five minutes of life, respectively. The patient had an uneventful recovery with resolution of her laboratory abnormalities within five days.

## Discussion

PRES and eclampsia

The literature first described PRES in 1996. The syndrome is characterized by clinical and radiographic findings, including headache, altered mental status, seizures, and vision loss with reversible subcortical vasogenic edema in the posterior cerebral white matter [8]. Conditions associated with PRES include hypertension, eclampsia, pre-eclampsia, autoimmune disorders, renal disease, and cytotoxic medications. Although the pathophysiology is not fully understood, there are several proposed mechanisms. The leading theory suggests that acute increases in mean arterial pressure cause autoregulation failure, ultimately leading to blood-brain barrier dysfunction and vascular leakage of plasma and macromolecules into the cerebral white matter [9]. This mechanism, however, does not explain cases of PRES in the absence of elevated blood pressure or with MAPs below 150mmHg [9]. Endothelial dysfunction from various endogenous or exogenous toxins, such as excessive circulating cytokines and chemotherapeutic drugs, creating cerebral edema is another proposed mechanism [10].

Pre-eclampsia and eclampsia are both pregnancy-related hypertensive disorders presenting after 20-week-gestation. Pre-eclampsia includes hypertension (SBP >140mmHg or DBP >90mmHg) accompanied by one or more of the following: proteinuria (>300mg/day), maternal organ dysfunction (acute kidney injury, liver involvement, neurologic complications, or hematologic complications), and uteroplacental dysfunction (fetal growth restriction, abnormal uteroplacental blood flow by Doppler) in the absence of other causative conditions [11]. Eclampsia is characterized by seizures in addition to the criteria for pre-eclampsia. Like PRES, the exact etiology of these conditions is unclear, although the placenta likely plays an integral role. The similarities in clinical and laboratory data of pre-eclampsia/eclampsia and PRES indicate a shared pathologic milieu. A study involving 110 patients demonstrated a 100% prevalence of radiologic evidence of PRES in patients diagnosed with eclampsia [4]. In another study, 92.3% of patients diagnosed with eclampsia demonstrated MRI-confirmed PRES [2]. Furthermore, the study found PRES in 19.2% of patients with pre-eclampsia and concomitant neurologic symptoms [2]. These studies demonstrate that PRES may be a primary component of nervous system injury in pre-eclampsia/eclampsia. Neurologic symptoms including headache, visual complaints, and seizures in pre-eclampsia/eclampsia are not new associations, but only recently has MRI identified vasogenic edema as one of the pathophysiologic factors.

Anesthetic management of patients with PRES and eclampsia

There is a paucity of literature on the anesthetic implications of PRES, which is distinctly different from the plethora of clinical experience, data, and recommendations concerning anesthetic management of pre-eclampsia/eclampsia, HELLP syndrome, and other comorbid conditions. Anesthetic considerations for pre-eclampsia/eclampsia include the potential for coagulopathy, hemorrhage, hemodynamic instability, severe intravascular volume depletion, renal/hepatic impairment, and airway edema [12]. While considering the risk/benefit of neuraxial and general anesthesia, the patient’s examination, laboratory studies, and physiologic derangements typically determine the safest anesthetic plan. Unlike pre-eclampsia/eclampsia, the choice of anesthetic technique and its effect on outcome in PRES is less clear.

The optimal anesthetic technique in PRES is uncertain due to multiple case reports directly implicating anesthesia as the sole cause of PRES. A temporal relationship and lack of other identified inciting causes have implicated anesthetics in several cases of PRES. In one report, the patient had a general anesthetic followed by a spinal with intrathecal morphine. The authors speculated that the intrathecal morphine was the cause of the patient’s PRES [5]. In another case involving a patient with PRES and eclampsia, the patient underwent general anesthesia and remained intubated for several days post-operatively due to the inability to control convulsions with speculation that the anesthetic contributed to this prolonged course [6]. Additionally, there is some evidence that dural puncture may be responsible for the development of PRES. In one case, a patient undergoing thoracic spine surgery developed PRES after sustaining a dural tear [7]. These cases suggest that exposure to anesthetic agents or techniques involving dural puncture may be a risk factor for development of PRES; however, the evidence is anecdotal at this point and requires further exploration.

## Conclusions

More information and an increasing number of reported cases are required to determine the safest anesthetic options in patients with PRES. First, clinicians must be aware of PRES as a cause of neurological symptoms in pre-eclampsia and eclampsia. Second, careful risk/benefit analysis while considering confounding conditions, physical exam, laboratory testing, and patient physiology should guide the choice of anesthetic. The pathophysiology of PRES with brain injury, vasogenic edema, and the potential risk of increased intracranial pressure further complicate anesthetic planning. These two unique cases add to the body of information and highlight the paucity of experience managing this condition during pregnancy. Furthermore, the advancement of technologies such as MRI has only recently shed light on this new finding, making it likely that clinicians will start to recognize and diagnose more cases of PRES in the future.
